# Identification and Functional Characterization of Two Putative Pheromone Receptors in the Potato Tuber Moth, *Phthorimaea operculella*

**DOI:** 10.3389/fphys.2020.618983

**Published:** 2021-01-25

**Authors:** Xiaoli He, Yajie Cai, Jinglei Zhu, Mengdi Zhang, Yadong Zhang, Yang Ge, Zengrong Zhu, Wenwu Zhou, Guirong Wang, Yulin Gao

**Affiliations:** ^1^Institute of Insect Sciences, Key Laboratory of Biology of Crop Pathogens and Insects of Zhejiang Province, Key Laboratory of Molecular Biology of Crop Pathogens and Insects, Ministry of Agriculture, State Key Laboratory of Rice Biology, Hangzhou, China; ^2^State Key Laboratory for Biology of Plant Disease and Insect Pests, Institute of Plant Protection, Chinese Academy of Agricultural Sciences, Beijing, China

**Keywords:** Phthorimaea operculella, pheromone receptor, *Xenopus* oocytes, pheromone communication, sexual pheromone

## Abstract

Pheromones are a kind of signal produced by an animal that evoke innate responses in conspecifics. In moth, pheromone components can be detected by specialized olfactory receptor neurons (OSNs) housed in long sensilla trichoids on the male antennae. The pheromone receptors (PRs) located in the dendrite membrane of OSNs are responsible for pheromone sensing in most Lepidopteran insects. The potato tuber moth *Phthorimaea operculella* is a destructive pest of Solanaceae crops. Although sex attractant is widely used in fields to monitor the population of *P. operculella*, no study has been reported on the mechanism the male moth of *P. operculella* uses to recognize sex pheromone components. In the present study, we cloned two pheromone receptor genes *PopeOR1* and *PopeOR3* in *P. operculella*. The transcripts of them were highly accumulated in the antennae of male adults. Functional analysis using the heterologous expression system of *Xenopus* oocyte demonstrated that these two PR proteins both responded to (*E*, *Z*)-4,7–13: OAc and (*E*, *Z*, *Z*)-4,7,10–13: OAc, the key sex pheromone components of *P. operculella*, whilst they responded differentially to these two ligands. Our findings for the first time characterized the function of pheromone receptors in gelechiid moth and could promote the olfactory based pest management of *P. operculella* in the field.

## Introduction

The potato tuber moth, *Phthorimaea operculella* (Lepidoptera: Gelechiidae), is one of the main pests affecting potatoes around the world (Rondon, 2020). It reduces potato production either *via* mining and damaging leaves in fields or *via* burrowing and destroying tubers in storage. The sex pheromone glands of *P. operculella* females produce a blend of odors that can influence the behavior of males. Two major pheromone components trans-4, cis-7-tridecadienyl acetate ((*E*, *Z*)-4,7–13: OAc) and tran-4, cis-7, cis-10-tridecatrienyl acetate ((*E*, *Z*, *Z*)-4,7,10–13: OAc) have been identified from this blend (Roelofs et al., 1975; Persoons et al., 1976). The structures of these components are quite similar, with the only difference in their double bond numbers. In field trapping studies, *P. operculella* males flew to lure sources containing either single or mixed compounds, while (*E*, *Z*, *Z*)-4,7,10–13: OAc attracted more male moths than (*E*, *Z*)-4,7–13: OAc, indicating the differentiated recognition of these components in male antennae. Moreover, when both compounds were present the number of moths attracted also increased significantly. While pheromone-based technologies have been widely used as part of sustainable control strategies, it remains unclear how these two pheromones are detected by *P. operculella*.

Insects use pheromones for mate recognition, and these chemical cues are mainly perceived by olfactory sensory neurons (OSNs), which are housed in olfactory sensilla on the antenna (Laurent, 1999; Steinbrecht, 1999). Pheromone detection is mediated by the heteromeric ligand-gated ion channels in the cell membrane of OSN dendrites. These channels are formed by the combination of specific pheromone receptors (PRs) and the olfactory co-receptor (Orco; Sato et al., 2008). Previous studies indicated that PRs belong to the olfactory receptor (OR) family, and they are thought to constitute a monophyletic clade in the phylogeny of insect OR for a long time (Liu et al., 2018b). However, recent studies found that except classical clade, there are multiple PR clades that have evolved independently (Yuvaraj et al., 2018). For example, Bastin-Heline et al. (2019) found SlitOR5, a PR from the cotton leafworm *Spodoptera littoralis*, grouped with a novel PR clade. With a typical structure of seven transmembrane domains, PRs are more abundantly expressed in male antennae (He et al., 2014; Zhang and Löfstedt, 2015; Ke et al., 2017), whilst some PRs are also found in female antennae and other tissues such as the wings and ovipositor (Chang et al., 2015; Li et al., 2020).

In Lepidopteran insects, PR was first functionally characterized in *Bombyx mori* using the heterologous expression system of *Xenopus* oocyte (Sakura et al., 2004), after that more than 60 PRs have been studied in over 30 moth species across 10 families by using different systems, such as in *Xenopus* oocytes (Wang et al., 2011), HEK293 cells (Forstner et al., 2009), transgenic *Drosophila* (Montagné et al., 2012), and more recently, in specific insects *via* CRISPR/Cas techniques (An et al., 2020). These PRs are predominately identified from moths causing crop damages, such as *Manduca sexta* (Große, 2010), *Spodoptera exigua* (Liu et al., 2013a), *Spodoptera litura* (Zhang et al., 2015a), *Plutella xylostella* (Sun et al., 2013; Liu et al., 2018b), *Ostinia furnacalis* (Liu et al., 2018a), *Athetis lepigone* (Zhang et al., 2018), *Sesamia inferens* (Zhang et al., 2015b), and *Athetis dissimilis* (Liu et al., 2019; Guo et al., 2020). Based on these studies, PRs were further grouped into three types: (1) narrowly tuned to a single component of the sex pheromones; (2) tuned to one component with high specificity, but still sensitive to other components at higher doses; and (3) broadly tuned to a wide range of pheromone components (Liu et al., 2018b).

Sex pheromone components have been characterized in *P. operculella* and other gelechiid insects and widely used in the management of pests, while the molecular mechanism of sex pheromone component perception is still largely unknown (Persoons et al., 1976). In the present study, we cloned two pheromone receptor genes *PopeOR1* and *PoprOR3* in *P. operculella*. Their transcripts were highly accumulated in the antennae of male adults. Their expression patterns were measured in different tissues of male and female adults by using the quantitative real-time PCR (qPCR). The function of these two candidate PRs in the detection of the key pheromones (*E*, *Z*)-4,7–13: OAc and (*E*, *Z*, *Z*)-4,7,10–13: OAc were further characterized using the heterologous expression system of *Xenopus* oocyte. Our results provide insights into the mechanism of pheromone perception in a gelechiid insect.

## Materials and Methods

### Animals

*Phthorimaea operculella* used for this study were collected from a suburban area in Yunnan Province in 2017 and the larvae were reared on potatoes at 26 ± 1°C on a 16:8 h (light/dark) photoperiod cycle and 70 ± 5% relative humidity at the Institute of Plant Protection, Chinese Academy of Agricultural Science (Beijing, China). The adults were fed with a 10% honey solution for three generations.

### RNA Extraction and cDNA Synthesis

Total RNA was isolated from tissues using Trizol Reagent (Invitrogen) according to the manufacturer’s protocol. Total RNA was dissolved in RNase-free water and gel electrophoresis was used to verify its quality. The concentration of RNA was determined by NanoDrop-2000 (Thermo Scientific, Waltham, MA, USA). cDNA was synthesized from 1 μg of total RNA using a RevertAid Frist Strand cDNA Synthesis Kit (Fermentas, Vilnius, Lithuania). The cDNA products were stored at −20°C until use.

### Gene Cloning

Two candidate PR genes, *PopeOR1* and *PopeOR3*, and the *Orco* gene (*PopeOrco*) were identified from the transcriptome of *P. operculella* (unpublished data). Their full length cDNAs were cloned with specific primers (Supplementary Table 1) designed by primer5.0 (PREMIER Biosoft International, CA, United States). The open-reading frames (ORFs) of these three genes were predicted using the ORF Finder.^1^ The PCR reaction was performed in a 50 μl system containing 25 μl of *TransStartPfu* PCR SuperMix (TransGen Biotech, Beijing, China), 22 μl of ddH_2_O, 1 μl of cDNA template, and 1 μl of forward and reverse primers (10 μM). The PCR conditions were: 95°C for 2 min; 35 cycles of 98°C for 10 s, 55°C for 30 s, 72°C for 1.4 min; 72°C for 10 min. The PCR products were verified on a 1.2% agarose gel, and the band was recovered and purified by an AxyPrep™ DNA gel extraction kit (YMbio, Beijing, China). Purified fragments were cloned in the pEASY®-Blunt3 cloning vector (TransGen Biotech, Beijing, China) and then transformed into *Trans5α* chemically competent cells (TransGen Biotech, Beijing, China). The transformants were incubated on LB-Agar plates containing 100 μg ml^−1^ of ampicillin. The target DNA products were sequenced by Novogene (Beijing, China).

### Phylogenic Analysis and Sequence Analysis

To construct the phylogenic tree, OR genes from *H. virescens*, *S. littoralis* (Walker et al., 2019), *M. sexta*, and *B. mori* were used, and the MEGA7 program was used for phylogenic analysis (Tamura et al., 2011). The phylogenic tree was constructed by the neighbor-joining method with a bootstrap test using 500 replications. The Genbank accession numbers for all the OR genes used are shown in Supplementary Table 2, and all the *P. operculella* OR proteins used for the phylogenetic analysis are presented in Supplementary Material.

Transmembrane domains of the two candidate PRs were predicted by TMHMM Server Version 2.0,^2^ and the amino acid sequences of PopeOR1 and PopeOR3 were aligned by the DNAMAN 8.0 software (Lynnon Biosoft, San Ramon, CA, United States).

### Tissue Specific Expression Profiles of Two Candidate Pheromone Receptor Genes

To determine the tissue expression profiles of the two candidate PR genes, female antennae (FA), male antennae (MA), heads without antennae (H), thoraxes (T), abdomens (AB), legs (L), wings (W), and genitalia (G) from 3-day-old unmated adult moths were collected between the 6th and 8th hours of the dark period, and were immediately frozen in liquid nitrogen and stored at −80°C. Total RNA was extracted and cDNA was synthesized as mentioned above. The qPCR analysis was conducted using an ABI Prime 7,500 Detection System (Applied Biosystem, United States). The qPCR reaction was performed in a 20 μl system containing 10 μl of SYBR Green PCR Master Mix (Biomed, Beijing, China), 0.4 μl of each primer (10 μM), 0.4 μl of ROX Reference Dye II, 1 μl of cDNA template, and 7.8 μl of nuclease-free water. The thermal cycling parameters were: 95°C for 1 min, 40 cycles of 95°C for 10 s, 55°C for 5 s, and 72°C for 15 s. DNase were used to eliminate the DNA contamination of the RNAs samples. The actin gene was used to standardize the target gene expression (Sun et al., 2013). For each tissue, three biological replicates were measured with three technical replicates for each replicate, gene expression levels were analyzed using the 2^−ΔΔCT^ method (Livak and Schmittgen, 2001). The sequences of the primer pairs used in this analysis are listed in Supplementary Table 1. The SPSS 20.0 software (IBM, Endicatt, NY, United States) was used for data analysis, the statistical comparison of the expression of the PRs was assessed using one-way ANOVA followed by Tukey’s honest significant differences (HSD) test (*p* < 0.05), data were presented as mean ± SEM.

### Vector Construction and cRNA Synthesis

Primers containing the Kozak consensus sequence and restriction enzyme cutting site (*Apa* I and *Not* I) were designed to amplify the open-reading frame (ORFs) of *PopeOR1*, *PopeOR3*, and *PopeOrco*. The products were then cloned into pT_7_T_s_ vectors with the primers listed in Supplementary Table 1 (Wang et al., 2011). The extracted plasmids were linearized by digestion with *Sam*I, and used as templates to synthesis cRNAs by using T7 polymerase of the mMESSAGE mMACHINE®T7 Kit (Thermo Fisher Scientific, Waltham, MA, United States). The purified cRNAs were diluted with nuclease-free water at a concentration of 2 μg/μl and stored at −80°C until use.

### Pheromone Components

The pheromones (*E*, *Z*)-4,7–13: OAc and (*E*, *Z*, *Z*)-4,7,10–13: OAc were purchased from Nimrod Inc. (Changzhou, China) with 95% minimum purity. The stock solution was prepared in dimethyl sulfoxide (DMSO) at 1 M concentration and stored at −20°C. Prior to the two-electrode voltage clamp electrophysiological recording experiments, the stock solutions were diluted with Ringer’s buffer (96 mM of NaCl, 2 mM of KCl, 5 mM of MgCl_2_, 0.8 mM of CaCl_2_, and 5 mM of HEPES; pH=7.6) into the concentration of 10^−4^ M Ringer’s buffer containing 0.1% DMSO was used as the negative control. All chemicals were freshly prepared for the experiments.

### Receptor Expression in *Xenopus* Oocytes and Two Electrode Voltage Clamp Electrophysiological Recordings

Each of the two candidate PRs were co-expressed with the PopeOrco in *Xenopus* oocytes for 3–4 d, and the ligand sensitivity was detected using a two electrode voltage-clamp recording as previously reported (Lu et al., 2007; Wang et al., 2010). Healthy, matured *Xenopus* oocytes (stage V-VII) were treated with 2 mg/ml of collagenase in washing buffer (96 mM of NaCl, 2 mM of KCl, 5 mM of MgCl_2_, 5 mM of HEPES; pH 7.6) for 1–2 h at room temperature. Equal amounts of PR and Orco cRNA (27.6 ng) were microinjected into the oocytes (Wang et al., 2011). The oocytes were then incubated at 18°C for 4–7 d in 1× Ringer’s solution (96 mM of NaCl, 2 mM of KCl, 5 mM of MgCl_2_, 0.8 mM of CaCl_2_, and 5 mM of HEPES; pH=7.6) supplemented with dialyzed horse serum (5%), tetracycline (50 μg/ml), streptomycin (100 μg/ml), and sodium pyruvate (550 μg/ml). Whole-cell currents were recorded from the injected *Xenopus* oocytes in an OC-725 two-electrode voltage clamp amplifier (Warner Instruments, United States) at a holding potential of −80 mV. Oocytes were exposed to a concentration series of different pheromone components from low to high with an interval between exposures that allowed the current to return to baseline. To avoid the sequential effect of two sex pheromone components on the candidate PRs, the experiment was repeated by reversing the order of component stimulation. Oocytes containing PopeOR1/Orco and PopeOR3/Orco were injected with 1× Ringer’s buffer solution containing 0.1% DMSO to be used as negative control, respectively. All experiments were repeated 5 times on different oocytes. The Digidata 1440A and pCLAMP 10.2 software were used to collect and analyze the data (Axon Instruments Inc., United States). Dose-response curves were obtained and analyzed using GraphPad Prism 5 (GraphPad Software Inc., United States). A statistical comparison of the response of the oocytes to the candidate ligands was assessed using student’s *t*-test with the SPSS 10.0.1 software (IBM, Endicatt, NY, United States).

## Results

### Gene Cloning and Phylogenic Analysis

**Figure 1:**
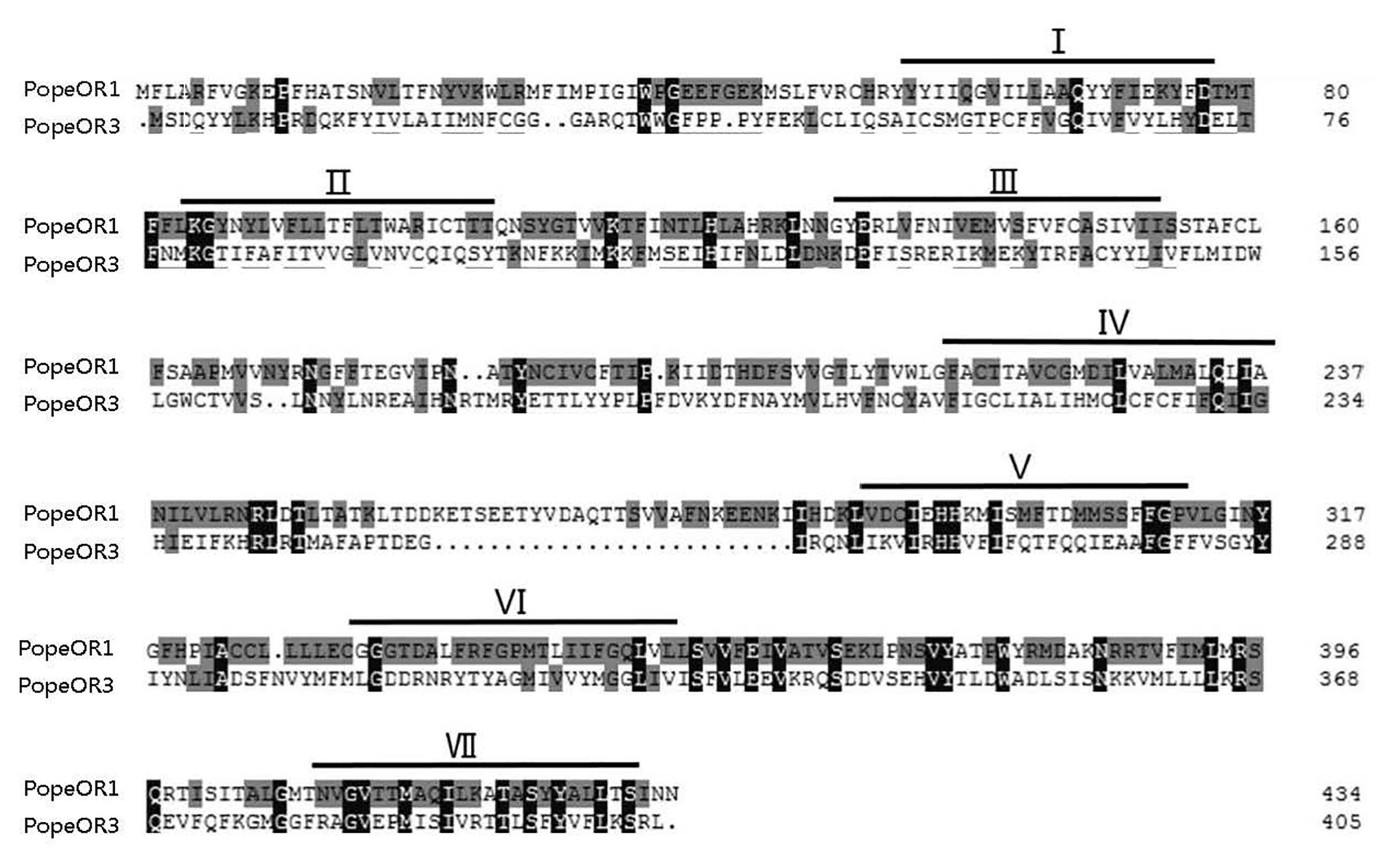
Alignment of the amino acid sequences of the two candidate PRs in *Phthorimaea operculella*. Identical amino acids are marked with gray and black shading. Predicted seven transmembrane domains (TMD1-TMD7) are indicated by bold lines.

**Figure 2:**
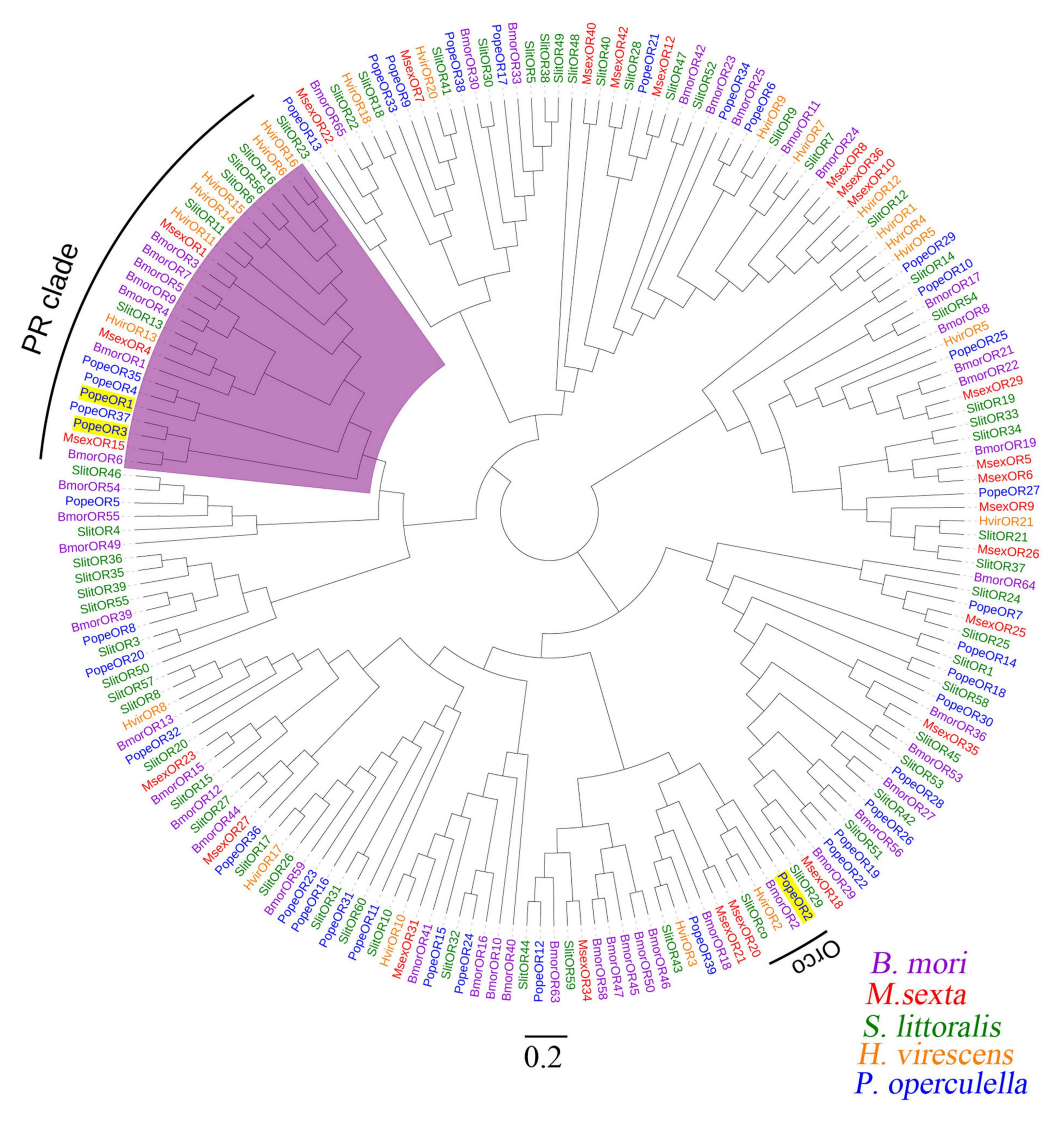
An unrooted neighbor-joining tree of the two candidate PRs of *P. operculella* together with the ORs from four other Lepidopteran species; *B. mori*: *Bombyx mori* (purple), *M. sexta*: *Manduca sexta* (red), *S. littoralis*: *Spodoptera littoralis* (green), and *H. virescens*: *Heliothis virescens* (orange) was constructed. The clade in purple indicates the pheromone receptor clade. The two candidate PRs and Orco of *P. operculella* are marked with yellow shading.

The full-length sequences of the three candidate PR genes were cloned, based on the nomenclature of ORs in other Lepidopteran insects, they were named *PopeOR1*, *PopeOR2*, and *PopeOR3*. In addition, *PopeOR2* was likely a pheromone co-receptor gene of *P. operculella*, and was further named *PopeOrco*. The lengths of the complete ORF region of *PopeOR1*, *PopeOR3*, and *PopeOrco* were 1,302, 1,215, and 1,419 bp, which encode 434, 405, and 473 amino acid residues, respectively. Consistent with other insect ORs, the predicted PopeOR1 and PopeOR3 proteins contain seven putative transmembrane domains (Figure 1) with a predicted extracellular C-terminus and an intracellular N-terminus. Phylogenic analysis showed that these two PRs were grouped into the same branch with PRs from other Lepidopteran species, and this branch was independent from other ORs which may mainly respond to general environmental odors. In the phylogenic tree, PopeOR1 and PopeOR3 clustered with PRs of other Lepidopteran insects including BmorOR1, BmorOR3, BmorOR6, HvirOR11, HvirOR13, and SlitOR11, SlitOR13. The PopeOrco gene (*PopeOR2*) clustered into the Lepidopteran Orco group with HvirOrco, SlitOrco, and BmorOrco (Figure 2).

### Expression Profiles of the Candidate PR Genes

**Figure 3:**
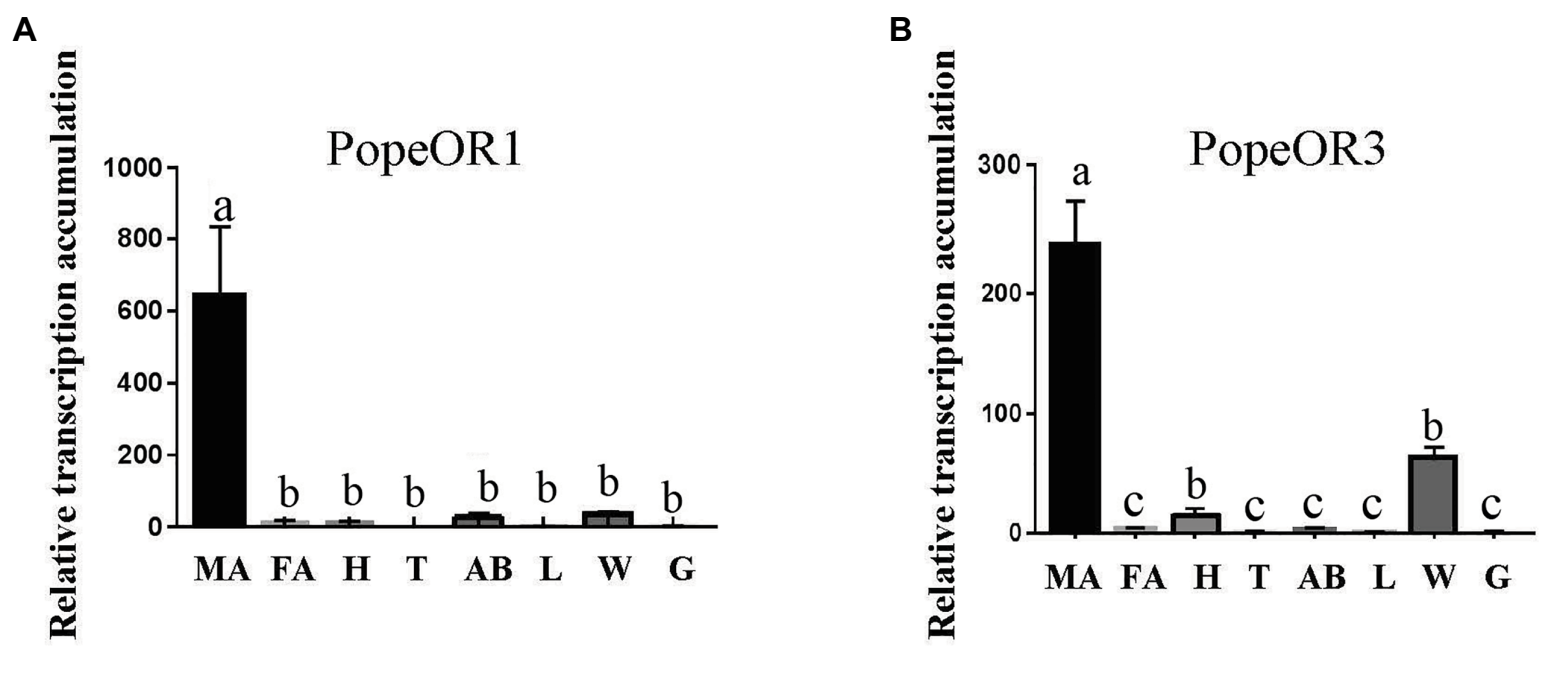
Expression profiles of *PopeOR1* **(A)** and *PopeOR3* **(B)** genes in different tissues. MA, male antennae; FA, female antennae; H, heads (without antennae); T, thoraxes; AB, abdomens; L, legs; W, wings, G, genitalia. Error bars indicate standard error of three biological replicates. Different letters within the same figure mean that the values are significantly different under one-way ANOVA followed by Tukey’s honest significant differences (HSD) test (*p* < 0.05).

The qPCR was performed to evaluate the transcription levels of two candidate PRs in different tissues of adults (Figure 3). As expected, the transcripts of both PR genes were highly accumulated in the male antennae. The transcript of *PopeOR1* was not expressed in other tissues including heads (without antennae), thoraxes, abdomens, legs, wings, and genitalia. For *PopeOR3*, its transcript was also hardly detectable in heads (without antennae), thoraxes, abdomens, legs, and genitalia, but not in the wings.

### Functional Characterization of the Two Candidate PRs in the *Xenopus* Oocyte System

**Figure 4:**
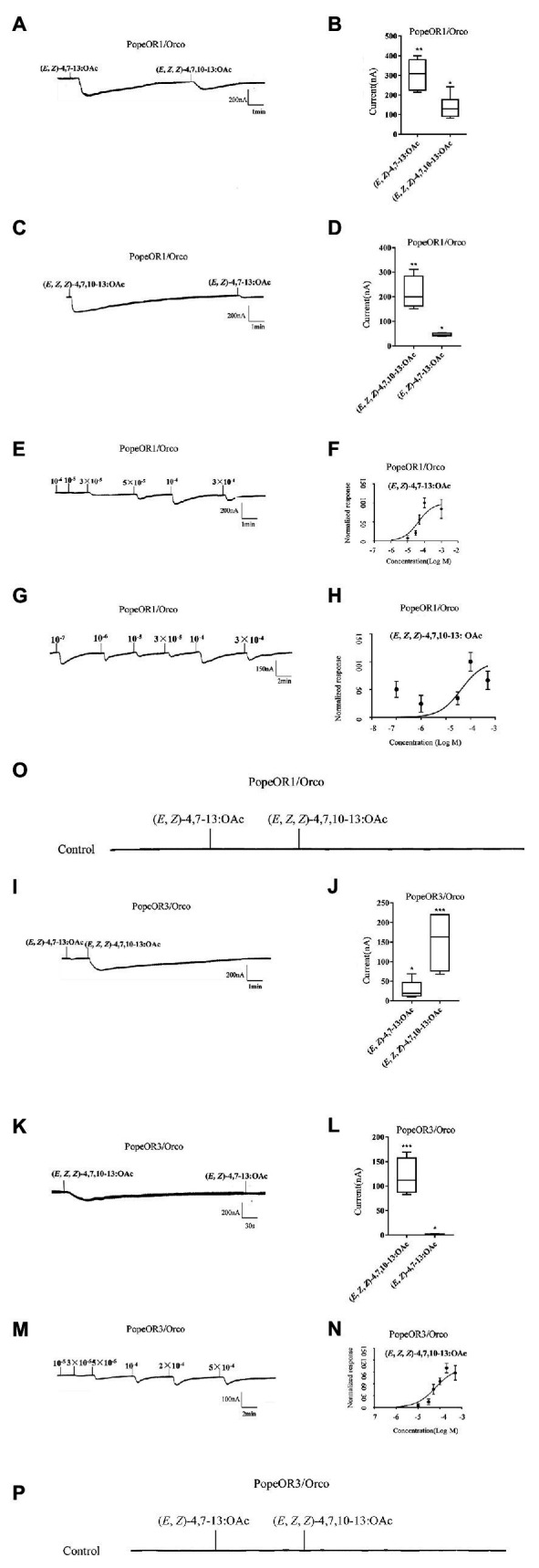
ContinuedFunctional characterization of PopeOR1 and PopeOR3 in *Xenopus* oocytes. **(A,I)** Inward current responses of PopeOR1/Orco and PopeOR3/Orco expressed *Xenopus* oocytes in response to a 10^−4^ mol/L solution of tested compounds. **(B,J)** Boxplots of tested compounds response profile of PopeOR1/Orco and PopeOR3/Orco expressed *Xenopus* oocytes. **(C,K)** Inward current responses of PopeOR1/Orco and PopeOR3/Orco expressed *Xenopus* oocytes in response to a 10^−4^ mol/L solution of tested compounds (reverse stimuli order). **(D,L)** Boxplots of tested compounds response profile of PopeOR1/Orco and PopeOR3/Orco expressed *Xenopus* oocytes (reverse stimuli order). **(E)** PopeOR1/Orco expressed *Xenopus* oocytes stimulated by a series of concentrations of (*E*, *Z*)-4,7–13: OAc. **(F)** Dose-response curves of PopeOR1/Orco expressed *Xenopus* oocytes to (*E*, *Z*)-4,7–13: OAc. Responses were normalized by setting the maximal response to 100. EC_50_ values were calculated to be 7.3 × 10^−5^ M. **(G)** PopeOR1/Orco expressed *Xenopus* oocytes stimulated by a serious of concentrations of (*E*, *Z*, *Z*)-4,7,10–13: OAc. **(H)** Dose-response curves of PopeOR1/Orco expressed *Xenopus* oocytes to (*E*, *Z*, *Z*)-4,7,10–13: OAc. Responses were normalized by setting the maximal response to 100. EC_50_ values were calculated to be 3.9 × 10^−5^ M. **(M)** PopeOR3/Orco expressed *Xenopus* oocytes stimulated by a range of concentrations of (*E*, *Z*, *Z*)-4,7,10–13: OAc. **(N)** Dose-response curves of PopeOR3/Orco expressed *Xenopus* oocytes to (*E*, *Z*, *Z*)-4,7,10–13: OAc. Responses were normalized by setting the maximal response to 100. EC_50_ values were calculated to be 8.9 × 10^−5^ M. Error bars indicate SEM (*n* = 6). **(O,P)** Negative control of PopeOR1/Orco and PopeOR3/Orco expressed *Xenopus* oocytes stimulated by tested compounds. Significance is indicated by asterisk.

The heterologous expression system of *Xenopus* oocyte and the voltage clamp recording technique were used to explore the function of the two candidate PRs. Both PopeOR1/PopeOrco and PopeOR3/PopeOrco were successfully activated by two pheromone components (*E*, *Z*)-4,7–13: OAc and (*E*, *Z*, *Z*)-4,7,10–13: OAc. PopeOR1/PopeOrco responded to (*E*, *Z*)-4,7–13: OAc and (*E*, *Z*, *Z*)-4,7,10–13: OAc with the responses of 313.7 ± 28.26 and 137.6 ± 19.22 nA, respectively (Figures 4A,B). For PopeOR3/PopeOrco, a strong response was observed when bound to (*E*, *Z*, *Z*)-4,7,10–13: OAc (155.7 ± 20.26 nA), while weak responses were found when bound to (*E*, *Z*)-4,7–13: OAc (29.41 ± 6.87 nA; Figures 4I,J). By reversing the order of the stimulation from the two components, the PopeOR1/Orco showed decreased responses on (*E*, *Z*)-4,7–13: OAc with a current value of 45.4 ± 3.207 nA, but enhanced responses on (*E*, *Z*, *Z*)-4,7,10–13: OAc with a current value of 215.5 ± 34.07 nA (Figures 4C,D), while changing the order of the supply of components had no impact on the sensitivity of PopeOR3/PopeOrco to them (Figures 4K,L). Oocytes injected with the buffer did not respond to any of the test compounds (Figures 4O,P).

In the dose-response comparison experiments, PopeOR1/PopeOrco responded to (*E*, *Z*)-4,7–13: OAc at the concentration of 10^−6^ M, and the response peak occurred at 10^−4^ M, with an EC_50_ value of 7.3 × 10^−5^ M (Figures 4E,F); PopeOR1/PopeOrco responded to (*E*, *Z*, *Z*)-4,7,10–13: OAc at the concentration of 10^−7^ M, and the response peak occurred at 10^−4^ M, with an EC_50_ value of 3.9 × 10^−5^ M (Figures 4G,H). PopeOR3/PopeOrco responded to (*E*, *Z*, *Z*)-4,7,10–13: OAc at the concentration of 10^−6^ M and the response peak occurred at 2 × 10^−4^ M, with the calculated EC_50_ of 8.9 × 10^−5^ M (Figures 4M,N).

## Discussion

Pheromone communication is widely used by Lepidopteran insects to find cognate individuals of the opposite sex (Vogt, 2005). Of all the protein families involved in pheromone perception, PRs play critical roles in determining the specificity and sensitivity of the recognition of the chemical mating signals (Liu et al., 2018b), which is considered an important basis for interspecies isolation and intraspecies choice (Groot et al., 2006). In the current study, we identified and characterized two candidate PRs from *P. operculella*, an important pest of the Solanaceae crop.

The expression analysis suggested that both the two PR genes in *P. operculella* were almost only expressed in male antennae. The males of Lepidopteran insects are the main receivers for pheromone cues. It is therefore reasonable to see the sex-specific expression of PR genes in *P. operculella* and many other Lepidopteran species (Krieger et al., 2004; Sakura et al., 2004; Wang et al., 2011; Liu et al., 2013b, 2018a). Moreover, it is interesting to see that *PopeOR3* is also expressed in the wings of male adults. A recent study into *Helicoverpa assulta* found that an odorant receptor gene, *HassOR31* which was highly expressed in the ovipositor rather than in antennae, was related to the determination of oviposition in host plants in females. This finding reveals that some ORs located outside the antenna might also have a functional role *in vivo* (Li et al., 2020). Therefore, further studies are needed to uncover the function of *PopeOR3* in the wings of *P. operculella*.

The heterologous expression in *Xenopus* oocytes and electrophysiological recording were widely used in investigating the function of insect PRs. In the phylogenic tree, PopeOR1 and PopeOR3 clustered with the PRs from other insects, including BmorOR1, BmorOR3, HvirOR6, and SlitOR6, whose functions in pheromone detection have been confirmed (Sakura et al., 2005; Wang et al., 2011; Cheng et al., 2017), indicating their potential roles as PRs. When co-expressed with PopeOrco, both PopeOR1 and PopeOR3 showed strong responses to the (*E*, *Z*, *Z*)-4,7,10–13: OAc, whereas only PopeOR1 had strong responses to (*E*, *Z*)-4,7–13: OAc. Thus, it is likely that PopeOR1 is more sensitive to female-produced pheromones than PopeOR3. Moreover, we found that PopeOR1 had a higher affinity to (*E*, *Z*, *Z*)-4,7,10–13: OAc (with an EC_50_ value of 3.9 × 10^−5^ M) than to (*E*, *Z*)-4,7–13: OAc (with an EC_50_ value of 7.3 × 10^−5^ M). A previous field study found that sole sex pheromones can attract males, and (*E*, *Z*, *Z*)-4,7,10–13: OAc was more attractive to *P. operculella* males than (*E*, *Z*)-4,7–13: OAc (Persoons et al., 1976). Our findings may partially explain this phenomenon. Despite the fact that single sex pheromone compounds can attract males, a field study revealed that when (*E*, *Z*)-4,7–13: OAc and (*E*, *Z*, *Z*)-4,7,10–13: OAc are mixed with a ratio of 1:4, the combination showed the highest attraction (Persoons et al., 1976). This implied that in the field the two pheromone compounds should stimulate the OSN together simultaneously and that co-stimulation of the two sex pheromone components would enhance the overall response of the neurons. A previous study on the PRs of noctuid moth *Spodoptera littoralis* indicated that two different PRs can detect the same pheromone compound with a different sensitivity (de Fouchier et al., 2015). In this study, we noticed that PopeOR1 and PopeOR3 showed different affinities to the same sex pheromone compound (*E*, *Z*, *Z*)-4,7,10–13: OAc. One possible reason is that these two PRs might be located on different sites of the male antennae, which contributes to their distinct affinities to the single same sex pheromone compound. Another possibility is that these two receptors are expressed by two different types of OSNs, in which case they are able to show different affinities for the same sex pheromone compound.

Although we identified the roles of PopeOR1 and PopeOR3 in the detection of two key pheromones *in vitro*, since we only tested two pheromone compounds in the present study, we supposed that these PRs might also detect other pheromone components untested here. Further studies in testing a wider range of pheromone compounds on this insect and also the identification of other candidate PR genes in the genome of *P. operculella* could provide more information on the pheromone detection of this pest. Moreover, while the *Xenopus* oocytes system is widely used to analyze the function of PRs, recent findings suggested that PRs were more sensitive to pheromones when pheromone binding proteins were present (Chang et al., 2015). It might be interesting to see the *in vivo* biological functions of PopeOR1 and PopeOR3 by using techniques such as CRISPR/Cas in *P. operculella*.

## Data Availability Statement

The original contributions presented in the study are included in the article/Supplementary Material, further inquiries can be directed to the corresponding authors.

## Ethics Statement

The animal study was reviewed and approved by Ethics Committee of Institute of Insect Sciences, Key Laboratory of Biology of Crop Pathogens and Insects of Zhejiang Province, Key Laboratory of Molecular Biology of Crop Pathogens and Insects, Ministry of Agriculture, State Key Laboratory of Rice Biology, Zhejiang University.

## Author Contributions

WZ, GW, and YuG conceived and designed the experiments. XH and JZ wrote the manuscript. XH, YC, JZ, MZ, YZ, and YaG performed the experiments. WZ, GW, and YuG provided valuable suggestions and helped to revise the manuscript. All authors discussed the results and approved the final manuscript.

### Conflict of Interest

The authors declare that the research was conducted in the absence of any commercial or financial relationships that could be construed as a potential conflict of interest.

## Footnotes

^1^ www.ncbi.nlm.nih.gov

^2^ www.cbs.dtu.dk/services/TMHMM/
