# Corrigendum: Identification and functional characterization of two putative pheromone receptors in the potato tuber moth, *Phthorimaea operculella*

**DOI:** 10.3389/fphys.2022.994614

**Published:** 2022-08-19

**Authors:** Xiaoli He, Yajie Cai, Jinglei Zhu, Mengdi Zhang, Yadong Zhang, Yang Ge, Zengrong Zhu, Wenwu Zhou, Guirong Wang, Yulin Gao

**Affiliations:** ^1^ Institute of Insect Sciences, Key Laboratory of Biology of Crop Pathogens and Insects of Zhejiang Province, Key Laboratory of Molecular Biology of Crop Pathogens and Insects, Ministry of Agriculture, State Key Laboratory of Rice Biology, Hangzhou, China; ^2^ State Key Laboratory for Biology of Plant Disease and Insect Pests, Institute of Plant Protection, Chinese Academy of Agricultural Sciences, Beijing, China

**Keywords:** phthorimaea operculella, pheromone receptor, xenopus oocytes, pheromone communication, sexual pheromone

In the published article, there was an error in the **Funding** statement. The funding statement for the Key Research and Development Program of Zhejiang Province was displayed as “Grant No. 2018C04G2011264”. The correct Funding statement appears below.

Funding

“the Key Research and Development Program of Zhejiang Province (Grant No. 2019C04007)”

The authors apologize for this error and state that this does not change the scientific conclusions of the article in any way. The original article has been updated.

