# Supplementary material for: Identification and Functional Characterization of Two Putative Pheromone Receptors in the Potato Tuber Moth, Phthorimaea operculella
Source: Front Physiol. 2021 Jan 25;11:618983. doi: 10.3389/fphys.2020.618983 (PMC7868389; doi:10.3389/fphys.2020.618983)
Supplement: Supplementary file 1 [file Table_1.docx]

Table S1 The primers used in this study.

| Name | Primer sequences (5′-3′) | | | Application | |
| --- | --- | --- | --- | --- | --- |
| PopeOR1 | | F: ATTCAATTCTGCCCGAAAGA | | For gene clone | |
|  |  | R: ATTACAGCATCGTCTCAAAC | |  | |
| PopeOR3 | | F: ATGTCTGATCAGTACTATTTAAAACATC | |  | |
|  |  | R: TTACAACCTTGACTTTAAAAACACA | |  | |
| PopeOrco | | F: ATCATACCGTCTTCGGGTCG | |  | |
|  | | R: ACGGGAAAATGGGTCGTTCG | |  | |
| PopeOR1 | | F: ATGGCTTGGTTTTGCATGCA | | RT-qPCR | |
|  | | R: TCCGTCAGTTTCGTTGCAGT | |  | |
| PopeOR3 | | F: GCCGTCTTCATTGGATGTTT | |  | |
|  | | R: CGAAAGCCATCGTTCGTAAT | |  | |
| Actin | | F: CAGACAGCAGCTTCATCCAC | |  | |
|  | | R: CGCCACATGATTCCATACCC | |  | |
| PopeOR1-ApaⅠ | | | F: TCA*GGGCCC*GCCACCATGTTTTTGGCAAGATTCGTCGG | | cDNA synthesis |
|  | | | R: TCA*GCGGCCGC*TTAATTGTTGTTTATAGAAGTGAGCAGT | |  |
| PopeOR3-ApaⅠ | | | F:TCA*GGGCCC*GCCACCATGTCTGATCAGTACTATTTAAAACATC | |  |
|  | | | R: TCA*GCGGCCGC*TTACAACCTTGACTTTAAAAACACA | |  |
| PopeOrco-ApaⅠ | | | F: TCA*GGGCCC*GCCACCATGATGACCAAAGTGAAGACGC | |  |
|  | | | R: TCA*GCGGCCGC*TTATTTGAGTTGTACCAACACCATG | |  |

Italic sequences (*GGGCCC* and *GCGGCCGC*) are the restriction sites of *Apa*Ⅰ and *Not*Ⅰ, respectively; and the underlined sequences are protective bases and Kozak sequences that are used to ensure the stability of gene amplification.

Table 2 Species and GenBank accession no. in this study.

| Protein name | Accession number | Protein name | Accession number |
| --- | --- | --- | --- |
| *BmorOR1* | NP_001036875.1 | *BmorOR38* | NP_001103477.1 |
| *BmorOR2(BmorOrco)* | NP_001037060.1 | *BmorOR39* | NP_001116807.1 |
| *BmorOR3* | NP_001036925.1 | *BmorOR40* | NP_001166608.1 |
| *BmorOR4* | NP_001036926.1 | *BmorOR41* | NP_001091787.1 |
| *BmorOR5* | NP_001036927.1 | *BmorOR42* | NP_001091818.1 |
| *BmorOR6* | NP_00103693.1 | *BmorOR44* | NP_001166607.1 |
| *BmorOR7* | NP_001106227.1 | *BmorOR45* | NP_001104798.1 |
| *BmorOR8* | NP_001157209.1 | *BmorOR46* | NP_00115549.1 |
| *BmorOR9* | NP_001116805.1 | *BmorOR47* | NP_001104818.1 |
| *BmorOR10* | NP_001104819.1 | *BmorOR49* | NP_001166614.1 |
| *BmorOR11* | NP_001166604.1 | *BmorOR50* | ACH73301.1 |
| *BmorOR12* | NP_00110484.1 | *BmorOR53* | NP_001166615.1 |
| *BmorOR13* | NP_001166603.1 | *BmorOR54* | NP_001166616.1 |
| *BmorOR14* | NP_001166602.1 | *BmorOR55* | NP_001166612.1 |
| *BmorOR15* | NP_001091789.1 | *BmorOR56* | NP_001166617.1 |
| *BmorOR16* | NP_001104832.2 | *BmorOR57* | NP_001159625.1 |
| *BmorOR17* | NP_001157210.1 | *BmorOR58* | NP_001166618.1 |
| *BmorOR18* | NP_001166895.1 | *BmorOR59* | NP_001166611.1 |
| *BmorOR19* | NP_001091785.1 | *BmorOR60* | NP_001155301.1 |
| *BmorOR21* | NP_001104831.1 | *BmorOR61* | NP_001166619.1 |
| *BmorOR22* | NP_001166613.1 | *BmorOR63* | NP_001166620.1 |
| *BmorOR23* | NP_001166606.1 | *BmorOR64* | NP_001166621.1 |
| *BmorOR24* | NP_001155300.1 | *BmorOR65* | NP_001166622.1 |
| *BmorOR25* | NP_00110483.1 | *HvirOR1* | CAD31850.1 |
| *BmorOR27* | NP_001166893.1 | *HvirOR2(HvirOrco)* | CAD31851.1 |
| *BmorOR29* | NP_001166894.1 | *HvirOR3* | CAD31852.1 |
| *BmorOR30* | NP_001091786.1 | *HvirOR4* | CAD31946.1 |
| *BmorOR33* | NP_001103623.1 | *HvirOR5* | CAD31947.1 |
| *BmorOR36* | NP_001166892.1 | *HvirOR6* | CAD31948.1 |
| *BmorOR37*  *MsexOR1*  *MsexOR4*  *MsexOR5*  *MsexOR6*  *MsexOR7*  *MsexOR8*  *MsexOR9*  *MsexOR10*  *MsexOR12*  *MsexOR15*  *MsexOR18*  *MsexOR20*  *MsexOR21*  *MsexOR22*  *MsexOR23*  *MsexOR25*  *MsexOR26*  *MsexOR27*  *MsexOR29*  *MsexOR31*  *MsexOR34*  *MsexOR35*  *MsexOR36*  *MsexOR40*  *MsexOR42* | NP_001104799.1  CUQ99387  CUQ99388  CUQ99389  CUQ99390  CUQ99391  CUQ99392  CUQ99393  CUQ99394  CUQ99396  CUQ99398  CUQ99401  CUQ99402  CUQ99403  CUQ99404  CUQ99405  CUQ99407  CUQ99408  CUQ99409  CUQ99410  CUQ99412  CUQ99414  CUQ99415  CUQ99416  CUQ99417  CUQ99418 | *HvirOR7*  *HvirOR8*  *HvirOR9*  *HvirOR10*  *HvirOR11*  *HvirOR12*  *HvirOR13*  *HvirOR14*  *HvirOR15*  *HvirOR16*  *HvirOR17*  *HvirOR18*  *HvirOR19*  *HvirOR20*  *HvirOR21* | CAD31853.1  CAD31949.1  CAD31950.1  CAG38111.1  CAG38112.1  CAG38113.1  CAG38114.1  CAG38115.1  CAG38116.1  CAG38117.1  CAG38118.1  CAG38119.1  CAG38120.1  CAG38121.1  CAG38122.1 |
