# Supplementary material for: Identification and Functional Characterization of Two Putative Pheromone Receptors in the Potato Tuber Moth, Phthorimaea operculella
Source: Front Physiol. 2021 Jan 25;11:618983. doi: 10.3389/fphys.2020.618983 (PMC7868389; doi:10.3389/fphys.2020.618983)
Supplement: Supplementary file 2 [file Table_2.docx]

All *Phthorimaea opercullela* OR proteins used in this study.

>PopeOR1

MFLARFVGKEPFHATSNVLTFNYVKWLRMFIMPIGIWPGEEFGEKMSLFVRCHRYYYIIQGVILLAAQYYFIEKYFDTMTFFLKGYNYLVFLLTFLTWARICTTTQNSYGTVVKTFINTLHLAHRKLNNGYERLVFNIVEMVSFVFCASIVIISSTAFCLFSAAPMVVNYRNGFFTEGVIPNATYNCIVCFTIPKIIDTHDFSVVGTLYTVWLGFACTTAVCGMDILVALMALQLIANILVLRNRLDTLTATKLTDDKETSEETYVDAQTTSVVAFNKEENKIIHDKLVDCIEHHKMISMFTDMMSSFFGPVLGINYGFHLIACCLLLLECGGGTDALFRFGPMTLIIFGQLVLLSVVFEIVATVSEKLPNSVYATPWYRMDAKNRRTVFIMLMRSQRTISITALGMTNVGVTTMAQILKATASYYALLTSINNN

>PopeOR2

MMTKVKTQGLVTDLMPNINVMRMCGHFVFNYLPEDGGMSILLRKAFASVNAFFIVVQYICMGINMAKYAEEVNELTANSITVLFFAHTLIKLLFFALNSKNFYRTLAVWNQSNSHPLFTESDARYHQIAVSKNRKLLYFICGSTLLSVVCWITITFFGESVYFVMDKETNETLTEPAPRLPLKAWYPFNAMHGTMYIVAFAFQVYWLVFAMMIPNVLDVLFCSWLVFACEQLQHLKAIIKPLMELSASLDTHRPNTAELFRASSTEKSEKIPEMTDADIRGIYSTQQDFGMTLRGAGGRLQNFGNNGTNPNNLTQKQEMLVRSAIKYWVERHKHVVRLVESIGDTYGTALLFHMLVSTITLTLLAYQATKIDGLNVYAFSTIGYLSFTLAQVFHFCIFGNRLIEESSSVMEAAYSCQWYDGSEEAKTFVQIVCQQCQKAMSISGAKFFTVSLDLFASVLGAVVTYFMVLVQLK

>PopeOR3

LWTVFELLRDKMSDQYYLKHPRDQKFYIVLAIIMNFCGGGARQTWWGFPPPYFEKLCLIQSAICSMGTPCFFVGQIVFVYLHYDELTFNMKGTIFAFITVVGLVNVCQIQSYTKNFKKIMKKFMSEIHIFNLDLDNKDEFISRERIKMEKYTRFACYYLIVFLMIDWLGWCTVVSLNNYLNREAIHNRTMRYETTLYYPLPFDVKYDFNAYMVLHVFNCYAVFIGCLIALIHMCLCFCFIFQIIGHIEIFKHRLRTMAFAPTDEGIRQNLIKVIRHHVFIFQTFQQIEAAFGFFVSGYYIYNLIADSFNVYMFMLGDDRNRYTYAGMIVVYMGGLIVISFVLEEVKRQSDDVSEHVYTLDWADLSISNKKVMLLLLKRSQEVFQFKGMGGFRAGVEPMISIVRTTLSFYVFLKSRL

>PopeOR4

MKTRKTLDVPSDDNEDVLSCKYVKLLRRYLWLLRLWPGEEFGEKVPFLIKVHGNYVQFQIVLVLVAQLIYIHKYYDTMNFFLKGYTYLLFLLSIVTFFRLFVTTLKSYGTIVKTLFNTLHLANRKLDEKFEKQVYIYVEKISYISTLTLIIMSYIGVSFFNIAPLYLNYKNGYFSGQQKENSTYNCIVCITIPKLTDSHDFSFFSTIFTMYITYPCTISVCGIDILLILMAFQIIGNMLVLRNRLETLSAKKEAITNDIIIDETQEDEQSSFVLFNEEENKLIHVKLVDCIKHHTTIIMFAEMMSSLFGPVLGLNYAFHLAACCLLLLECAGGTDAIFRFGPLTVIIFSQLILLSVVFEIVATVSEKLKNSVYFMPWERMDSRNRRTVYIMLARAQTPIRVNALGLANVGVTTMAQILKMTASYYALLNSISK

>PopeOR5

QIKMGLFEYMKKKVLYVKFRLTDNSVDSMLWLINIVPSLAGFSLKKERITIFTPFWIIHLSLLFYVYGVGNLVYQKYYAQEAGDFIKSFVNVSLLVLIANNSYWFLTQRPLLQEIIKEVKQSDSMARDSPEFHKKHEKLMATVYRIVLIFYAVNYGNASCIYLPHRSDVDNEFSMTPCVGIRPLSAFPKRQICSIILCLQELSIMTVVLNYQALLLLFVDHTSAMYKLLSDEMITFNQPDVDDDYVKRKLPSLLQRHILCLSIVNKFKLLFSMPIGINFLSNAACISLFFYLPLPEWLGFIPILVYCFLVFFLYCFLGQRLIDSAEAFERAVYGCGWEKFDVKIQKTIYLMLMNAQAKIEILAADIIPVNIYTFATTIQFMFKFVTVMKF

>PopeOR6

VNAAMTGKGLLELTYPAPAITHCFLANIKAYYYLKSEHQVNDLISSLRKLQAMTVSGDLSAEVEKELQERMTYLTKATNIQYWLNVVSIAGFAILPFVTMGMSYAETGKIELMMPFVLLLPFDVFNKFIYPFLYLLDMFAATMIVFMVLGPDSLFYACGTFIQIQFRYLRYDLERVIARNPTILRLQQQIAVCIKRHKDLIRCVSLMEHIYAKSTLFNVVSSSMLICLTGFNLTASPSKILMMPFLGFVFLVSGQIYLFCYFGDNIMRSSLAISDGVYNCQWYYADKKVKKDLLFILLRAQKPCKLTAYGFSDINLSTFCRILSSAWSYFALLSTVNRTNVATTT

>PopeOR7

MRVLNFLWTTLTPTQALNETSGPYETAFFEPVYRTLYLTGLSSSDKGLLYLAYSFTVKFLIVTFVGCELWYLFSMSEDLDEVIANINVTLIHLIAMYRYRSMIINGDIFKKLVMSTKSPHFDISTPRRRAILDFWLTNNERYFKLLLLLGTCTLAAWYVYPLVDDLDYNLVVTVRLPYDYAKPSYYPVTYAVVLIAFNYASYFVMVNDVIMQSHLMHLICQFMVLGDCFESIVEDCARDFLDLDQSALHRNKAFVLKYNNRLGDLVEQHKFILSNTLELRRILSVPMLYQMAASSMLICFAGYQVTATATSADITKFLMSLLYLGYNMFELFIFCRWCDEIKHQSEKIREAVYFSGWERVSLIPGVRARLMMVIARASRPMVLTAGGVYDLSLNSYGTIVKTSYSALSVLLRTQHD

>PopeOR8

MWFRFRKYGMGYCDLPTMLSNVTTMLKFLCLDIDPRNTKGVSFIFYIILTFTSTCYYYVYLISMIWFVFVRCIETGDLLAAIIVFSLGASSEISMTKLWCMFSERKNLRKLISSFLECDALVLPDSRFANHMKITLRTVKKRAITVWIVIIMNGIIYVAIPLVFLCFSKRRFMEDSFEFLGFDNMATMKTSPYFEFAFVINAVGVYVTCYPSANISALFIILAGYIEAQMLALAQELTFVWTDALKNYNNAHIITHFKDTNSTSTHNSESQELSGIGTKAWSDKEATSRTLSDIKHISTRTSIQRAFGPNIISEEIITQKERREINVYIHKRLAEIIKSHSMNINLLAKVEGVFRIAIAFEFLFLSIGLTAELLGDLGKTYIQLPFALMSVSIDCLIGQRIKDASMAFERAVYDSKWENFDVTNRKIILLMLRNSQKVMKLSAGSVATLTLECLMVVLKTTYSAYTTLRSTMN

>PopeOR9

MQSIIKYFVKEKKKDDEEVNEKDLSQDPAEKEFKSFPETYRIITFALSFGLMYPNPATRWFRFIGISVMLGTAIPLAVFILIDIYKCWLRKDIFEIIRHSTIVGPFLGGFLKQYFMVIFQDPANAIINEINRDYELYNRLEEDYKTIVRAHVNNCLVNSEKWWTVTVLTCVMIFPIMAATFTLYSFLFSDMPQKYMVHELNKPWDKDPEARFESPYYEIANFYMLYACCFYMLNFLGYDGFYGLAINHAVLKMQMYCKALQDAMQYEGKKVYERVVGVIYEQNRTFRYMDEIQNCFNLWLGIIFLATMIQLCNLLYHISAGNTLDVRYFIFIAGVTVHIYLPCHYSAKVKFASAAVSTSFYTCGWESARDQRIRMLLQLMIQRAQEPRAITAMGMMTFDMELFVQILQTSYSMYTLLSS

>PopeOR10

MAKKAAMASQESDEEYLDDPIQLIDLKQKRTMATGQKHDAGDTTYPIFELIDSDEDLYIEKTDNFSKKSLPRPKEVPITLPEQNDVVQFDIAHSTICAKFVVLYLQKKRIRVLLESFIEEDREYASLELERVAVKRAFRYCSAMQGTLYTTLFFYLVDGIRMHFKEGIPVRAEVVYYPSPEHHGVFVNILRVFVELHWWYIVSMMTCIDCLSVCSMVYLAYKYRCLGVYFAGLRQKLNENFGKMSVEAALKKYRDDFVVGIKLHEHALSYAKQIQHALGNLYSIQVIETMFLLVLALFKLVVNERSLTFLISSATYMTCIVVLLGGYMLAGGDITHEASLLPNAIYNSGWELTRGDKELRTLTVVALIRSQRPVIMTAFGVLNLSYSNFILVLRHSYSFFAVMY

>PopeOR11

ACEKYKSNMKQNSSCLATSITILKVTGTWFPDGLIRPWLYTSFAGVILLLFFVATVVAEIGYLFTVIGDTERIVEAAVLLLSHLVQGVKIMNICVRQGRIKRLIQFIDGPEFDKTDPKKVEILDKTVYLTTIIGQTIIACATVTAIFWCLVPGLKTELSLPLKTAYPFDISGYPILSVVICGINDTAVNYIVAGLMVLAAAHLDVLSEELNEIDAEGKKDNYQETIRRIKYHQSIIQYVNELAGIFGLPILCQFMATAMIVCMTLYKITVTSQPIELMTMIFYMICIFLELFMYCFPGDMIVNKSLSVAIAAAPATWTGDLRSRRALLLCCARAQRVLCVNAGSMFRVSLPTAAVVLRAAYSYYAVLQQKMDN

>PopeOR12

MKILIKNVNLSVSLSLTALRFVGFWSPEGLTETRRLLYNVYTGFSFMFLLGTYLIIQIVDLFNIWGNLPLMTGTAFVLFTNLAQAAKIANLLSKKRRILNVINEANDVLGAETNGEGMEIVKRCNREMIFLQVVYLTLTLITTTGWASSAEKGQLPLRAWYPYDTSKSPAYEITYLHQVGAINIAACLNVGKDTLVSALIAQCRCRLQLLGLSLRTLCRGLEMTDKLVYTPEQEAIAAERLRNCVKQHQAALEAAKEIQSCFSAPTFFQFFVSLVIICVTAFQLISQTGNLVRLFSMGTYLLNMMFQVFIYCYQGHHLSEESADVAGYAYKCPWYACSVRLRKMILVIMTRTRRVTKITAGGFTTLSLGSFMAIIKASYSLFTLLQQVTE

>PopeOR13

TAVMALILRKIRGFMEKENYDFDRPDIDLFNFHPQLCFFYMINGIFFNNRNSILRFFIPTLCSMAVFVGLGFELMFIHHGIDIKDYPFAAESFCYIMLLVTVPNLYTSVLMRRDLIIEQIDLINGDFLYIRNLGPRHRDSFTKGQLKIWRWCWTWLGLCFGAAFVYVLNGFGMLLYHTFFVTLDENSVRPLIFPFWLPHDDPYRTPNYEVFLFFEIWLIFIINQSFGAYIYIQFHILLHYINLIDMIILDIEVIFEGLDESVVELPMHDPRRMEVRGILNGRLKRIVYWHSAVFKSIDMLSSVYGPTLAYQVGFSAIIICLVAYQVVDSLEKGKVHIFFCLLLVEAFVQLWIPCYLGTLMKDKALSIADTCWNCGWERTDLGRMVCTDFLIIMLRAQKTLTIKFTGLPSLTLETFSSISSTAYSYFNMLRQSTKK

>PopeOR14

LNMGKKSNLEDTEKVHNYTHYLVLPLKFVACWDWFPKPTKEYQIIINNIYLALVLFVLSNLPIALTIHLYTEWEDIMESLEKIADGLPIVVSVAIVAYFASHKDQLYDLVEYMNKNFKFYSAKGLTNMTMRQSYNRAKNFAFVYTACTLFSVTMYVMLPIVVHLWTRTPLQLWVYKDAHGPFFAIMFLRQAVGQAFVGLAMGQLGVFFAANAILLCGQYDLLCCSLRNVRYTALLQRGVSHTAIAKAHSDIVSDERHNYIYSSAELKQSDYHYDGLLRDDFNGNITEFDIYSPAYDEATCAALQECARVCQAAAEYKQRFELFVSPLLALRVVQVTLYLCMLLYAATLKFEMITVEYLAAVALDIFVYCYFGNQIILQASRVSTAAYQSAWPSMGAKPRRMLLNILLANKRPVVVRAGRFLPMDLHTFVVIIKTSFSYYTLLVNVNENKSKH

>PopeOR15

NLYTRGPNAHSLWALIPLFDNSGPRIFPFKIWMPVNPEYSPDYELGYFYQMIAIYISACLFCGVDSVALCTMMFGCAQLEIIGDKLLMIRNVPLLSKEVKSNAERVKENTELLKDCIQHHQAVIKFIKLIEDTFHANIFFQLSGTVGIICIIGLRISVVDPHSVQFSSMINYMMTMLSQLFLYCWCGQELTIRSERLRQWLYQCPWYEQDTKFRRSLWIAMARMSKPIIFRAGHYIPLSRPTFIQILRSSYSYFAVLNQAKNK*

>PopeOR16

MEKPLSYLSIEPHFKTLRATGVFPLNDNDEVSKWKKIFYSLYTHFSFYLIPIYMLQQAVNVCKVKDDANKVMDTLFLLITNSDYVYKHAVFWRRYKEIEKLITIMKGPIFNQGGIEHEPILSQTARHSHVFLRIFNSAALSVCVLWVVYPIILFGQNNPVDFAIWLPFDYNENNKNFYAAVLYVYFQTSWLAINNTTMDATISFFLAQSKTQLSILRVDLESLVQRSKQEAKFSEEFYVNILQKRFRDRLLHYNEIVNFTEILQGIFSGPFLYQFMVSGWIICITAYRTLKMDPMSVEFASMILYLSCIIVQLFLFCHFGHEVNWESACVIDSAYYMDWLAFPIKLRKDLIIFMERLKRPLKPMGAFIPLSNSTFVSIMRSSYSFYALLKNTN

>PopeOR17

LTMDFDFNSIFKISLRAMRFNRTHPNIPRDGKWFLQFLVMHGVATFVVYLVAYSTICHDFKNNNFTQASENFIIICLYWIVTFCYFMMLWKLDSVRWLIDAAQKDFAAAKYLPAAEQEMLMGYALRGRWSCRLWLVISCCVATIFPLRSFCLMVYYSITDEFRLIPLYEVVYPEFLEKDRENLLSFVILFATMLWLCVYAASLYVALIPLGHIFMLHACGQIELLINRAGVLFPKINYNSSKAERDLKGIARGLQDVYSFVDHVQWSFQLIYELCLKSTTITMPLLVYELTKTTKHGEFNMEFFAITIGSVVLSGVPCFYSDQLMELSYNFRQTLYCSGWERHWDSRARTILLMMLVRTGRPAVIRSMFGNMSLRALGNVYHHSYAIYNLLNAMW

>PopeOR18

GQFLLLRKLTEGIAGRGRGYKLTSERDARAHYRIKRCHRTLKILDNSVVLLDNLVQNILGIYFLVATLTLCSVAMRLSTEEMSVMKVIPLVQYTGACLVQLFLFCYYGDMLLHRSSVGMGEGAFASAWWCLTPRLRREVLLLAAWQARSRRLRAGPFNRLDLPSFIQIVRAAYSYYAVLRK

>PopeOR19

RYVLLHIVATFFVISQYMELYVIRTDLDGVLTNLTISTLSILCVVKANTCVFWQSSWREVFDYVTEADKYERDTRDNVKGKILDNYTKYCRRVTYFYWILLFTTFVTVTHTPLFRFITSANYREELRNGTEPFPHIFSSWMPFDKYNSPGSWMTVVWHVLICVYGATIVAAYDTSMIVVLVFFGGKLDLLRERSKQMLGSGGHGVSDEHAAKMVQELHNVHVLLTKYSRLFNRLLSPVMFVYVLMCTLMLCASAFQLTSAQSTTQQFLMAEYLVFGTVQLFMFCWHSNGVVIKSEDLVYGPYESEWWTANVRQRKAIHLLGGQFRIRYMFNAGPFTDLTLATFITILKGAYSYYTLLTKGK

>PopeOR20

RLPLHYALVCTGVHIYSKTKWDSLLNLSHQLFNTFIAILVFIFTTGFIIVRTDDVFVWIEGFCIWITGVIIHITTGVALVFRKEFRQFVEEMALIDAMRKMPFIHHTLNNQYEEGKLKELKELVLGSQEKLLRYTNLLLKCYTTSVLSTGVLYITDAIYQMIVREDESLRLLGFEMWFPWKLDDFNVYIGTFIFHVYAGFFCAMGYPALQSTIVLLMGQIIRQLRILTYILENLDELVVEMCHKRDIKWQQYCTAVLSQSIDHYVMIKKFGNRLNRICRTFYLVLILDAMMLVCVCSVKIAISNKLSHDLIKYYMHEFFFTLMVFLFCFLGQQVQNECEKLENAVIEKWYIFDKKHRDNIKIFHVAVGQKMPILIFGSIPLSLPTFTWFIKTGMSFFTLAMSVLEE

>PopeOR21

AVIPTMSESSTEQAEKEIDGSLKLCTFCMRLIGLSFQPPKTATANLLQKLMLAISVLSICYHVFSEIVFIGLALSNSPKVEEVVPLFHTFGYGALSIAKVFVLWYKKDVFAQLIQELVEIWPVSPLSDEDQMIKNKSLIALRITSSCYFAANFLGIWFYNATPIAIYFYQVLVQGQSDAQVGFVWVSWYPFDKYEPFNHVAVYIFEMFAGQTCVWIMIGTDLLFSTMASHICLLLRLLQRRLESVANSEQKTPTECYKDIVDNIKLHQRLIRYCNDLEDAFAVSMLINYVLSSINICCVVFVIVLLEPFLAVSNKLFLASALIQIGMICWYGDDILHANSEIANSAYNSGWYKAHPRCRRALQFLMNRSQKPIAFTAMKFTNISLVTYSSILTRSYSYFALLYTMYSDK

>PopeOR22

MFLYVLMCTLTLCSSAFQITMAQSATQQFLMAEYLVFGTVELFMFCWHSNGVVIKSEDLVYGPYESEWWAANVRQRKAIHLLGGQFRIRYMFNAGPFTNLTLATFITILKGAYSYYTLLTKGK

>PopeOR23

MTPMLVVSREGGDGGSLRGAGSADTLAGRCVAPHLQCLRWCGFCRLAPDTKPMLRDSHYIYCLFAFSACLLYLVQECFYAYQERSDMDKLSRVMFLLLCHVTSAVKQVVFYMDTGRVDSLIREFDDPLFNQSWGARALTATADRARRLQRAYSGAALATCVLWLVFPVAQFLQNQPVEFAFWMPLSTRHPVGFLVVLLYSFYVTTLVGIANTTMDAFIGTVLYQCTTQMRILRTNLENLPDRAMEYSKAYGEDFETGLKSLFRDCLSHYQKICSITSLLQDLFGTAILVQFTIGGWILCMAAYKMISLNVLSIEFASMNLFIMCILTELFVYCYFGNELTYESARLADSLYACGWEGAEVAAGRRRWFARALLLAGERARRPLSVAAGRLVPLSLQTFLKILKSSYTFYAVLRQTK

>PopeOR24

GTPSSRSQRNRGALNVEDLYLNRAKFVMKFLGVWVPGDDETIFHKIYRIFMMSLQYMFLLFQIIYIIQVWGDLEAVSQASYLLFTQACLCFKVTVFQANMGLLRDLLARMNGDTFKPQSEEHEKTLKIQAARIKTLLLAFMVSSQTTCGLWALKPLFDDAGSRKFPFDMWMPVRPEQSPHYHVGYAFQLMTICMSAYMYFGVDSVALSMVIFGCAQIDIVKDKIMKIKSPLSKEIAQTDVKKVQDDNYKKLVECIVQHQAVISFTKLVEKAYHTYLVFQLTGSVGLICMSALRILVVDWKSMQFFSILTYLSVMISMLYVCCWCGHELTATSEDFHRVLYKCSWYEQDIKFKRALAFVLMRVNRPMVLRAGHYIPLSRQTFVAILRMSYSYFAVLNQAK

>PopeOR25

GRFVAMSVQDDRHKMKSYSILRTHCQYIWYVGTGNFWFEKGVVRADDTVAYRLFQVILFSTYIFMTIFEIMGISQDFPKDEHGDCVTFSVSHTIVNIKIFSILRQKNLIRKLNEKMVEICEEYESEDLMRNKYKIIKINIYAYFSIVYGTVLCFIFEGFRKMYEGTHFVTIVTYYPSYEDNSPLATAVRVLFTIILCILMMTMIFSVDSFTMMYLIMFKYKIITLKQYLEGIRKTFDKECKVNLRMASLNLKNGLIKAIVMHGALIKMCKDIDTAFGAVVTLQLCLSSGSVVSLLLQLALATDLPPVTTIKIIAFACALYFLLALFLCNAGDISYQAYLLSDAFFHSGWHLTESPRELLPMILMSTRQAQRPLVMKAFNMIEITYGLFLQVVRTTYSVFALFYAQNK

>PopeOR26

NKMLKSLLEKLENPKRPLLGPNVHALEFWGLLLPKNIFKRYFYLLMHFLVTVFTFTEYVDIYFIKNDLNLVLNNVKITILATMSVVKVTTFLTWQEKWRSIIEYVTRADMEQRNSKDDENKNQIITDYTKYCRKITYFYWSLMYTTVIIVVFTPIFKYLLSESYRVNVMNGTEPYTEVVSSYVPWSKETIPGYLAASLYQTYAATYGGGWITSFDTNAMVIMVFFRGELEMLRVDCRKIFGDGSKKVNDKVAMDRLKDLHRRHVELVKHANLFDSCLSPIMMLYMFVCSVMLCVTAYQITIETSAAQKFLTTEYLVFGVAQLFIYCWHSNDVLYTSQDLMLGPYESAWWTSPKLQKDLYILIEQFRKIIVFSAGPFAKLTVPTFIAILKGAYSYYTLLSKSHME

>PopeOR27

MNGLNKPPNRTRDYFQRISKVIYLMGAQDYWCEDLNLPSAVKLFHFCFMNFVNVASVALILSQWAVYFTDQKLTAKQSFDSKLFALAHPLSLGTVLITEKSRKKVSQLLQKLTIDLKEVHNDESVETAMMKKSKFVLKCYSTMIFMTAFLFGLEGFMMVKRGHTFVTMVTAWPDFEDTSTTASASRVIIYIVWCFLLIRLTSVFIMVIPLSICLSHQYKNLCSYFIKLNDIFKEDCSQEEKEIKYEKAFKVGIQLHSETMVCAHQLQELFGAVYGGQIIIKSTSVMAMMSSMANAERTLSHVVSTIGTASYSLISLGYLICYAGYTTVEAASLPSAIFFSGWQNCTKKTNTRIRPLVVIALLMSQRPVVLKCLGSVTLSFSSYVTIVKSCYSGFSVLY

>PopeOR28

QKWGMLQRKGFLSNFLFYFYFYALVYFVTQYIELWNLRNKQDEALKNLSYTLLSTVCYYKAGTLIIWKKRWIKIIDYVLDVETSQKSNTDRDVESLIHQYKKYSRIVTYLYWGLVGNTVVIVMLAPILAGFLSSQNRKRILSGADPYPEIMSSWVPFDKTGGAGYIASLIIQGSVYLWGGGFVAFYDSTAFVVMTFFAGQFKILKINCQKIFNDVKDLNNTSVQNRIKACYKQHDNLIKHCEILNELMSPVQFLYVIVCSIMFCASAIRVTSSEGSGLMQKLWLAEYLLALISQLFLYCWHSSEVYCCSHTLEYGVFSSAWWAADKNLRRNIILLHGKLRKSIYFSAGPFTILEISTFVAIVKGSYSYYTLLK

>PopeOR29

AMEKPDRPRRYLGLHYTVLRFLGLGWWHHPEEEDNRNFPGLYLYYTIVTELVWVVGFVGLETIDPFIGEKEMDRFMFSLSFVITHDLTVIKLYLFLFKNVELQDIVHTLEIKLQDFYQNYDKTRATIRTTRILTGSFIFFGWVTIGNANIYGAIQDLKWRVEVSQLNDTVPIPDRTLPQPIYIPWNYQKDISYIPTFILETVGLLWTGHIVMIIDTMVGTIILHMSSQFAIFQEALLSVYDRTVMQLYEGIREVNEKELEVKEDKLDLYEAVVKESYSKEEFEMALEKSLKKCFRHHQLLIGCVEKFGQTYSYGFMTQLLSSMAAICVVMVQVSQDASSFKSIRLITSLSFFVAMTIQLGLQCYTANEMTLEAGRVAEAVMKCKWERMPPRFRPLLKLMMMRAQRPLRLSAAGFANMDNACFLAIMKAAYSYYAVLSQRQDNGN

>PopeOR30

NSYIARCRRVVYFYWILCVAICSTIIFGPLVMIQQDEHGNCIYEHIINGFTPFDNQAPECYYTLFIEIIASNIMAFYLVGWNTLVWSIMIFFAGQLKIVQLYGMRLIEIGNNKKSHKNVAEFHRFYMSLVEHQKLFNSLISLSMFLYEVMTAINLSANIINIVQFSGDTSTLHSLLNSGACVLGCFIELLMLYWYGSKVGDESSLVSYATFSSSWVGADPQVQKEVALLALTTSKDIVFDAGPFHKMTLAAFVTILRASYTFFTLISTTK

>PopeOR31

SYNGYEAFRPHFDALARVGYFKLIPKSESSWKNSLHYVYRIAVWIIVGTYNLQHIIKAVIERHSTDQIVNTMFILLTTINTMMKQSAFNVRSRRIDQVIGVISGPVFAPQNPYHEKLMKANAVAMHRLLLFYISGIFVCGSLWTIFPLVNHALGEDVDFTAYFPFDTKRSPAFYLVVAYMNIFITFQAYGNVTMDCTIVSFYAQANIQLKILRYNLEHLADNVKEIAGGVKNVRAYTGYKDVDDENFNRVLQKRFTQCVVRHLEIKRFILEIESIFAGTLTFQLFVTAWVICMTVYKIVGLSLLSAEFLSMAMYLWCMLAQFFIYCYYGTQVRNESEYVIQSAFHANWVSLSVHFRRKLIILMENCKRPIIPRTAYVVPISLETYIAVIRASYTLFTFLDRK

>PopeOR32

PGSWNPRFYKYYSRVIIGTFIIIYNLLFTINFYFVDKNLDSVVQEMIFYFTEVACTSKCFTFLIMHDEIVKMLSILESNIFQPVSQNGINIICNAKKLNIKYWKFITGTSVSCNIAHVIIPVIAHIFLSTNLDLPVTSYSFLSDSFREKYNYLLYFYQSVGIHVVMWFNINIDTFILGVMILIIAQLDIIDEKLRNITDEDKSSEELVNLNDEEKEIQLMSYFNECIIHYDEVGKFVSLFQRIFSITLFMQFSMSSYIICVCLFRFTLPAPFQYRIFLATYMTCMIFQVMAPCWFGTQIIVKSMNLTKAVYACDWTPRSNRFKTNMRFFCTRAGQPLIIIGWKMFPMALTTFTAIMKSSYSAFALLRNMQSRQ

>PopeOR33

MVAIFIWDIWSSWLRRDIQNIVRHGTIIVPFFLVLTKTYVMYIQRQAAKEVIDEINYDHERLNYLPESYRPAVEEHFRNAHYGEKLWLTVVIHCVMIFPFASTVMMFYQYSVKSQPRKYMIHDVLLPFIEPEDRFETPYFEFIYLYMLYCCIILFLNMAGYDGFFGLAIRHACLKLRLCCIAMDDAMKRDDKDDMFEEIVEVIRAQCRVYKYVDTIQTTFNFWLGMIFLGTVVHVCNCMYQIMEGLGLDLKYLIFICGSVIHIYLPCNYASTLKSMSADSATLFYGCGWERVNDQRARYARLHDRARTTPSRHHRLQHTHFRYGAVCVYPPYIVFHVHASKTKGLETTSQKSRRTKEVLDITLHFL

>PopeOR34

RDIVNLEKDTLEKVEPKVIIERNVKIDTEAIERKIVEDSTWFLNFVIKLLCYSSTGVTAAFTTLPLMTMTYDFYKTGEWELSLPFLTKYFFDPFTRIMWPIVYLHQVWSTFIAAFNVFGVDTFLYAFCTYMHMHFSILCHRFEHVVSDSVVETRQRLKQLIKRHQELIELVNQVELMSSKSTLFNIITSSVLICLSAFNVTFMDDSATIIAFLTFLAMSLSQISLLCLFGDLLMRSSTKISEGIYKCRWYDTDPGVKRSIFLILIRAQKPCRLTAADFADLNLTAFTTILSRSWSYFALLRTVYK

>PopeOR35

VFVTIEKLSDVSILSIILISCAGCSSFCLAPMYLNYKNGFLTGQQKEDAVYNCLTCFTIPKLVDTHDFSVLSTIYCVYLAFIVTIAVIGMDMLLILMAFQIIGNLMIFINRVETLYASNDIIIKETYKDTQPASSQGFNNKGNEIIQEIISESKENNVFHITNENTHYEYKSFRPFNVEENKIIYEKLVNCVKHHSMIILFAKNMSNVFGSILGINYGFQLASCCLLLLECQGGFDAMIRFGPVTFIIFGQLILLSIIFEIIATTSEKLTTSVYCTPWERMDTRNRRTVQIMLVRAQRPISVTALGLTNVGVTTMAQILKTTASYYALLNSFNN

>PopeOR36

NFEVLPTNVEYPFFSVYNSPCYEIIYAHHVYYKPATCIIDGVMDTLLAAFVASAIGQIEILSYNIRNFAMVADRRRRRDIAANKYLGNSSEDYSKAVLRECIIHHNSIIKYVSMIESAFSLASALQFMLSVMVLCLIGIQFLSIEEPMSHPMQIVWMMIYLTCMLTEFFILCWFGDELIWKSIELRRAAFEAPWLDISPKTAMFIIIFLERCNRPLRVTAGKIFTLSLNTYTVLINWAYKAFAVMRNMKK

>PopeOR37

FWLDYNIHLCNIEDQDNFVKEERIKFEKLSRYSCYFLIAFLMTDGFGWTTILVVNNYLNRESIKNRTMQFETQLYYPIPFFESSYNFYAWLPVHIFNTYCVFIACIVIIIALTTNFMFVFQAIGHIKIFKYRMQTIYVESLKDEECFYKLIEIIKYHIFMINAFKEFEQTFGFFVSAYYLFNLTTDSYFVYMFMLGDDRNRFTYLGMFIVYMGSLIIMSFVLEKVKRQSDDISVRVYSINWLDMSIKNKKTFLLLLMRSQSVLQFTGMGGFRAGVAPMINIIKSTFSLYVLLKAKQS

>PopeOR38

RGKLVVYLWTTALFLYCIDAVLMPLMLMLYYLIFDEFKYVNTILLTLPPIFDDFNDDLRNFFVTVMTYSMMLYCSATYVAVFPQGLIYMLHACGRIDILKRKVLSLFPEDNVVPVDIHHNMKVIGKELQDIYDFVYDINDTSGVIYEVTLKVSAIIIPISALLIIEELKHTTVSLHFVILLAATLLICSLPCYYGNLLMTKSMELREAVFFCGWERVWHKPTRKSLIIIMTRIERAIAISSVFYVMNLETLADVFKTSHRLFSILSAAWTLEY

>PopeOR39

AIQGLVCSLMMYVCDQLIELQENLRSLKYSPETETEMREKFKQIVKKHVRLIEYSKEMDKIFQQFFIVQNLAVTVEMCLNAMMVTLVGLREKYLLVNFLACLGMALLNAYIYCYLGNELIIQSVGISVSAYETAWTSWPIDMQKNLLLVIRQAQKPISLSAGGIATMSIETYCQALYNGYSIFAVLYDAVN
